# Targeting PCSK9 to upregulate MHC-II on the surface of tumor cells in tumor immunotherapy

**DOI:** 10.1186/s12885-024-12148-2

**Published:** 2024-04-10

**Authors:** Hanbing Wang, Xin Zhang, Yipeng Zhang, Tao Shi, Yue Zhang, Xueru Song, Baorui Liu, Yue Wang, Jia Wei

**Affiliations:** Department of Oncology, Affiliated Hospital of Medical School, Nanjing Drum Tower Hospital, Nanjing University, No. 321, Zhongshan Road, 210008 Nanjing, China

**Keywords:** PCSK9, Bioinformatics analysis, Tumor immune microenvironment, Dendritic cells, Major histocompatibility complex-II

## Abstract

**Background:**

Proprotein convertase subtilisin/kexin type 9 (PCSK9), the last member of the proprotein convertase family, functions as a classic regulator of low-density lipoprotein (LDL) by interacting with low-density lipoprotein receptor (LDLR). Recent studies have shown that PCSK9 can affect the occurrence and development of tumors and can be used as a novel therapeutic target. However, a comprehensive pan-cancer analysis of PCSK9 has yet to be conducted.

**Methods:**

The potential oncogenic effects of PCSK9 in 33 types of tumors were explored based on the datasets of The Cancer Genome Atlas (TCGA) dataset. In addition, the immune regulatory role of PCSK9 inhibition was evaluated via in vitro cell coculture and the tumor-bearing mouse model. Finally, the antitumor efficacy of targeted PCSK9 combined with OVA-II vaccines was verified.

**Results:**

Our results indicated that PCSK9 was highly expressed in most tumor types and was significantly correlated with late disease stage and poor prognosis. Additionally, PCSK9 may regulate the tumor immune matrix score, immune cell infiltration, immune checkpoint expression, and major histocompatibility complex expression. Notably, we first found that dendritic cell (DC) infiltration and major histocompatibility complex-II (MHC-II) expression could be upregulated by PCSK9 inhibition and improve CD8^+^ T cell activation in the tumor immune microenvironment, thereby achieving potent tumor control. Combining PCSK9 inhibitors could enhance the efficacies of OVA-II tumor vaccine monotherapy.

**Conclusions:**

Conclusively, our pan-cancer analysis provided a more comprehensive understanding of the oncogenic and immunoregulatory roles of PCSK9 and demonstrated that targeting PCSK9 could increase the efficacy of long peptide vaccines by upregulating DC infiltration and MHC-II expression on the surface of tumor cells.

**Summary:**

This study reveals the critical oncogenic and immunoregulatory roles of PCSK9 in various tumors and shows the promise of PCSK9 as a potent immunotherapy target.

**Supplementary Information:**

The online version contains supplementary material available at 10.1186/s12885-024-12148-2.

## Introduction

Proprotein convertase subtilisin/kexin type 9 (PCSK9) is the ninth member of the proprotein convertase family [1] and is expressed mainly in the liver, lung, pancreas, and brain. One of its major functions is regulating blood lipid levels [2]. Studies have shown that gain-of-function and loss-of-function mutations in PCSK9 can lead to hypercholesterolemia and hypocholesterolemia, respectively [3, 4]. Moreover, emerging studies have shown that PCSK9 contributes to the occurrence and development of tumors. Therefore, it can be considered a potential target for treating an extensive spectrum of tumor types, including gastric cancer [5], liver cancer [6–9], small bowel cancer [10], lung cancer [11] and breast cancer [12]. Mechanisms of its pro-tumor effects include promoting tumor cell proliferation, apoptosis, invasion and migration by regulating antiapoptotic protein levels [13, 14], modulating the endoplasmic reticulum stress pathway [13], the mitogen-activated protein kinase (MAPK) pathway [5], the tumor necrosis factor-α (TNF-α) pathway [15] and the Bax/Bcl-2/caspase-9/caspase-3 pathway [6]. Furthermore, PSCK9 could facilitate the immunosuppressive tumor immune microenvironment (TME) by hampering the infiltration and functions of CD8^+^ T cells. On one hand, PCSK9 could downregulate the expression of major histocompatibility complex-I (MHC-I) molecules on the surface of tumor cells through lysosomal degradation and downregulate tumor-infiltrating cytotoxic T lymphocytes (CTLs) [16]. In addition, inhibiting PCSK9 can enhance the anti-tumor activity of CD8^+^ T cells by upregulating low-density lipoprotein receptor (LDLR) levels and promoting the interaction of LDLR with the T-cell receptor [17]. Thus, PCSK9 is a promising target for cancer immunotherapy and combinatorial treatment. However, no comprehensive analysis has been conducted to explore the role of PCSK9 in a pan-cancer perspective.

Our study revealed the correlations between PCSK9 expression and the clinical characteristics and prognosis, TME status, and MHC expression in pan-cancer patients. Then, we explored the anti-tumor activities of PCSK9 blockade and its regulatory effects on the TME in syngeneic mouse models. Our study comprehensively investigated the immunoregulatory role of PCSK9 in multiple tumor types and provided a theoretical basis for the application of PCSK9-targeted immunotherapy in tumor patients.

## Materials and methods

### Data sources and analysis tools

The data we used in this article were downloaded from the TCGA database. The analysis tools we used included TIMER (https://cistrome.shinyapps.io/timer/) and TIMER2.0 (http://timer.comp-genomics.org/).

## Cell lines and experimental mice

The mouse gastric cancer cell line MFC and melanoma cell line B16F10-OVA were routinely cryopreserved in our center. All cells were cultured in RPMI-1640 medium (MULTICELL, Canada) supplemented with 10% fetal bovine serum (Sijiqing, Hangzhou). The cells were incubated at 37 ℃ and 5% CO2.

SPF 615-line male mice and SPF C57BL/6 male mice were used for animal experiments. The mice used in the experiments were 4–6 weeks old males or females with weights of 18–22 g; the mice were purchased from Nanjing Cavens Biotechnology. The animals were kept in the SPF barrier system of the center, the temperature was maintained at 20–26 ℃, the humidity was maintained at 50–80%, and the mice were free to eat and drink with 12 h of light day and night. The use and operation of the experimental animals shall strictly follow the relevant regulations of the animal experiment management committee of this unit.

### PCSK9 expression and clinical characteristic analysis

To comprehensively analyze the mRNA expression of PCSK9 in tumors and adjacent normal tissues across 33 types of cancer in the TCGA cohort, we downloaded RNAseq data generated from the TCGA database, then analyzed and compared the RNASeq data of TPM format after log2 transformation. Notably, we analyzed paired and unpaired samples. The Mann–Whitney U test (Wilcoxon rank sum test) was used for statistical analysis, and the R package in R (version 3.6.3), mainly the GGplot2 package, was used to visualize the data.

We downloaded RNAseq and clinical data from the TCGA in HTSeq-FPKM format [18] and conducted log2 transformation. Then, we divided the tumor cases that retained clinical information into T1&T2 vs. T3&T4 groups, N0 vs. N1&N2&N3 groups, and Stage I & Stage II vs. Stage III & Stage IV groups and evaluated the expression level of PCSK9. We used Kruskal–Wallis test as the statistical method. Statistical significance was defined as follows: ns, *P* ≥ 0.05; *, *P* < 0.05; **, *P* < 0.01; ***, *P* < 0.001.

### Survival prognosis analysis

We used the survival package and survminer package to explore the correlation between PCSK9 expression and overall survival (OS), disease-specific survival (DSS), and progression-free interval (PFI) across 33 tumors in TCGA. The log-rank test was used as the statistical method. The analysis and visualization of receiver operating characteristic (ROC) curves were carried out using the pROC package and the ggplot package.

### Immune microenvironment analysis

We used the ESTIMATE package to determine the relationships between PCSK9 expression and stromal score, immune score, and ESTIMATE score for all tumor types in the TCGA cohort. Then, we analyzed immune infiltration via the TIMER database. We input “PCSK9” into the “Gene” module, selected “B Cell”, “CD8^+^ T Cell”, “CD4^+^ T Cell”, “Macrophage”, “Neutrophil”, and “Dendritic Cell”, selected all tumor types to obtain the P value and COR value via Spearman’s test, and generated a heatmap. Furthermore, we used TIMER2.0 to explore the correlation between PCSK9 expression and four immunosuppressive cell types, including M2 macrophages, myeloid-derived suppressor cells (MDSCs), regulatory T cells (Tregs) and cancer-associated fibroblasts (CAFs). In addition, we used the “correlation” module of the TIMER data analysis website to explore the relationship between PCSK9 expression and the expression of “PDCD1”, “CD274” and “CTLA4” in different tumor types. Finally, we used the Spearman analysis method to analyze the correlation between PCSK9 expression level with MHC-I and MHC-II expression levels in RNA-seq data from the downloaded TCGA database.

### In vitro mouse spleen cell treatment and coculture

6-week-old 615-line female mice were sacrificed via cervical dislocation after anesthetization with isoflurane (2.0% v/v) vaporized in oxygen and nitrous oxide (N_2_O) (50:50) using a universal-type anesthesia machine for small animals (R500IE; Reward Academy, China). Spleen cells were acquired from their spleens followed by red blood cell lysing and washing. For direct treatment assay, upon isolation, spleen cells were cultured in RMPI-1640 medium added by DMSO or PCSK9i (2 µM and 10 µM) for 48 h. For the tumor cell coculture assay, spleen cells were first cocultured in RMPI-1640 medium with IL-2 (20ng/ml, PeproTech) for 48 h, and MFC tumor cells were cocultured in RMPI-1640 medium with PCSK9i (10 µM). Then, spleen cells and MFC cells were cocultured in plates for 12 h and harvested for subsequent analysis. For the tumor-killing assay, MFC tumor cells were first cocultured in RMPI-1640 medium with PCSK9i (2 µM and 10 µM) for 48 h. Then, MFC cells were labeled by PB (1 µM), cocultured with spleen cells in plates for 12 h, and stained with PI (0.1 µg/ml) for 10 min before analysis.

### Western blot analysis of PCSK9 expression in cell lines

Western blot analysis was performed to evaluate the expression levels of PCSK9 in cultured cells. Cells were harvested and lysed in RIPA (NCM; WB3100) buffer containing ProtLytic Protease and Phosphatase Inhibitor Cocktail (NCM; P002). Protein concentrations were quantified using the BCA Protein Quantification Kit (Vazyme; E112-01). Equal amounts of protein (30 µg) were separated on a 10% SDS-PAGE gel and transferred onto a PVDF membrane. The membrane was blocked with 5% BSA in TBST for 1 h at room temperature, cut before hybridization with antibodies, and incubated with a rabbit anti-PCSK9 primary antibody (1:1000 dilution, Proteintech; 27882-1-AP) or GAPDH (1:1000dilution, Vazyme; MP102-02) overnight at 4 ℃. After washing, the specific bands of membrane were incubated with an HRP-conjugated goat anti-rabbit secondary antibody (1:5000 dilution) for 1 h at room temperature. The bands were visualized using an enhanced chemiluminescence (ECL) kit (NCM; P10060) and quantified via densitometry.

### Establishment and treatment of mouse models

Gastric cancer cell line MFC was selected in the logarithmic proliferative phase. The cells were collected after trypsin digestion and washing, and the final cell concentration was adjusted to 1 × 10^7^/mL. 615-line male mice (SPF grade) weighing 20–22 g were selected for subcutaneous tumor implantation. For the anti-tumor effect evaluation model, approximately 1 × 10^6^ / (100 µL) per mouse was subcutaneously (s.c.) implanted into the left groin of 615-line mice. MFC tumor-bearing mice were randomly divided into two groups, with 6 mice in each group, and treated with PCSK9 inhibitor PF-06446846 hydrochloride (MedChem Express, USA) or DMSO, respectively. The implementation day was recorded as Day 0 (D0), and the drug was given 48 h after tumor implantation (D2). PCSK9 inhibitor was administered intraperitoneally at 5 mg/kg, once every other day for 10 injections. The tumor volumes were measured every 2 days since Day 4. The long diameter was defined as a, and the transverse diameter perpendicular to the long diameter was defined as b. The subcutaneous tumor volume was calculated according to the following formula: tumor volume = a × b^2^ /2. Meanwhile, the mice’s body weights were weighed every 2 days, and their diet and activity were observed. For the CD8^+^ T cell functional status evaluation model, approximately 5 × 10^6^ / (100 µL) per mouse was subcutaneously implanted, and drugs were administered every day since Day 2 for 5 days.

B16F10-OVA tumor-bearing C57BL/6 mice were randomly divided into 4 groups of 6 mice in each group and were treated with DMSO, PCSK9 inhibitor, OVA long peptide, or PCSK9 inhibitor combining OVA long peptide, respectively. The day when the tumor was implanted was recorded as Day 0 (D0). The PCSK9 inhibitor was treated by intraperitoneal injection. 48 h after the tumor was seeded (D2), the drug was given. The dose of PCSK9 inhibitor was 5 mg/kg, and the drug was treated 8 times every other day. OVA long peptide was injected subcutaneously. After 72 h (D3) of the tumor induction, the drug was given at a dose of 10 µg per mouse. The tumor volumes were measured every 2 days since Day 4. The long and transverse diameters perpendicular to the long diameter were defined as a and b, respectively. The subcutaneous tumor volume was calculated as a × b^2^ /2. The body weights of mice were weighed every 2 days. The animals were observed for their diet, range of activity, and so on. At the endpoint, the animals were sacrificed through cervical dislocation after being anesthetized with isoflurane as mentioned above.

### Flow cytometry analysis

For in vitro analysis, cells were harvested from culture plates and made into single-cell suspension. For in vivo analysis, tumor tissues were harvested after the mice were sacrificed via cervical dislocation after being anesthetized with isoflurane as mentioned above, and cut into pieces in a six-well plate. RPMI1640 medium (2mL) containing 1 mg/mL type IV collagenase and 100U/ mL DNA enzyme was added to each well, and the samples were digested at 37 ℃ for 30–120 min. Then, the digestion was terminated with RPMI1640 and filtered with a 40 μm filter while grinding. After red blood cell lysing, the single-cell suspension was obtained. To measure the expression membrane markers, flow cytometry antibodies were stained for 30 min at room temperature and washed with PBS. Flowjo software was used to analyze raw data. Antibodies used were shown as follows: anti-mouse CD3e (PE-Cy7, #100,320), anti-mouse CD3 (FITC, #100,204), anti-mouse CD8 (Percp-Cy5.5, #100,732), anti-mouse CD25 (PE, #12-0251-83), anti-mouse CD69 (FITC, #104,506), anti-mouse CD107a (APC, #121,614), anti-mouse CD11c (FITC, #117,306), anti-mouse MHC-II (APC, #107,614), and anti-mouse CD103 (Percp-Cy5.5, #121,416).

### Statistical analysis

GraphPad Prism 9 software was used for statistical analysis of data and graphic representations. As for comparisons between two groups, unpaired t-test, log-rank test or Wilcoxon rank sum test were used as indicated. Regarding comparisons between more than two groups, one-way ANOVA was used as indicated. As to comparisons with double factors, two-way ANOVA was used as indicated. The error bars of data were presented as the means ± SEM. The p-value of less than 0.05 was considered to be statistically significant. ns, not significant.

## Results

### PCSK9 expression was elevated in tumor tissues and correlated with later stage of cancer patients

**Fig. 1:**
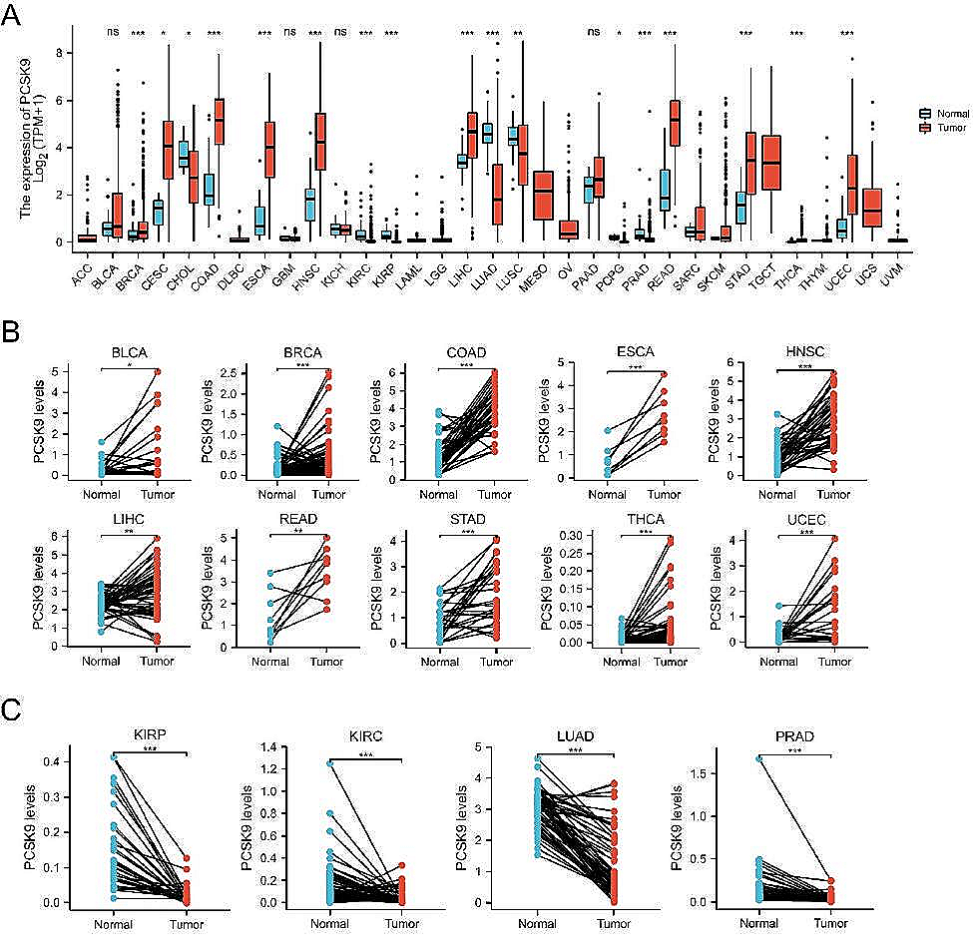
mRNA expression levels of PCSK9 in different tumor types. **(A)** Expression level of PCSK9 in unpaired samples among 33 tumor types. **(B)** The expression level of PCSK9 in paired samples in BLCA, BRCA, COAD, ESCA, HNSC, LIHC, READ, STAD, THCA, and UCEC. **(C)** Expression level of PCSK9 in paired samples in KIRC, KIRP, LUAD, and PRAD. ns *P* ≥ 0.05; *P < 0.05; **P < 0.01; ***P < 0.001

We systematically compared the mRNA expression levels of PCSK9 between normal tissues and tumor tissues across 33 tumor types in the TCGA cohort. The information on the 33 tumor types is summarized in Table S1. Overall, the expression of PCSK9 was higher in tumor samples than in normal samples among most tumor types, including breast invasive carcinoma (BRCA) (*P* < 0.001), cervical squamous cell carcinoma (CESC) (*P* < 0.05), colon adenocarcinoma (COAD) (*P* < 0.001), esophageal carcinoma (ESCA) (*P* < 0.001), head and neck squamous cell carcinoma (HNSC) (*P* < 0.001), liver hepatocellular carcinoma (LIHC) (*P* < 0.001), rectum adenocarcinoma (READ) (*P* < 0.001), stomach adenocarcinoma (STAD) (*P* < 0.001), thyroid carcinoma (THCA) and uterine corpus endometrial carcinoma (UCEC) (*P* < 0.001) (Fig. 1A). Furthermore, to exclude the influence conducted by differential numbers between tumor samples and normal samples, we analyzed PCSK9 expression in paired samples of available tumor types in TCGA. These results are consistent with the unpaired sample analysis except for those of CHOL and lung squamous cell carcinoma (LUSC) in which the expression of PCSK9 was not significantly different from normal tissues (Fig. 1B, C, Figure S1A).

Next, we explored the relationship between PCSK9 expression and TNM stage in 33 tumor types in the TCGA cohort (Figure S1, Table S2). In bladder urothelial carcinoma (BLCA) (*P* < 0.01), kidney renal clear cell carcinoma (KIRC) (*P* < 0.05), kidney renal papillary cell carcinoma (KIRP) (*P* < 0.01), THCA (*P* < 0.001) and diffuse large B-cell lymphoma (DLBC) (*P* < 0.01), the results showed that PCSK9 expression was higher in stage IV&III tumors than in stage I&II tumors, while the opposite outcome was observed in UCEC (*P* < 0.01) (Figure S1B, Table S2). We further investigated the correlation of PCSK9 expression and T or N stage. In BLCA (*P* < 0.01), KIRP (*P* < 0.001), STAD (*P* < 0.01), and THCA (*P* < 0.001), the expression level of PCSK9 was enriched in the T3&T4 subgroup than in the T1&T2 subgroup, while the opposite result was identified in mesothelioma (MESO) (*P* < 0.05) (Figure S1C). Among the KIRP (*P* < 0.05) and THCA (*P* < 0.001), the N1&N2&N3 group expressed higher PCSK9 than the N0 group (Figure S1D). In summary, PCSK9 expression was aberrantly higher in tumor tissues and related to later disease stages in most types of cancer.

### PCSK9 as a prognostic and predictive biomarker across multiple tumor types

**Fig. 2:**
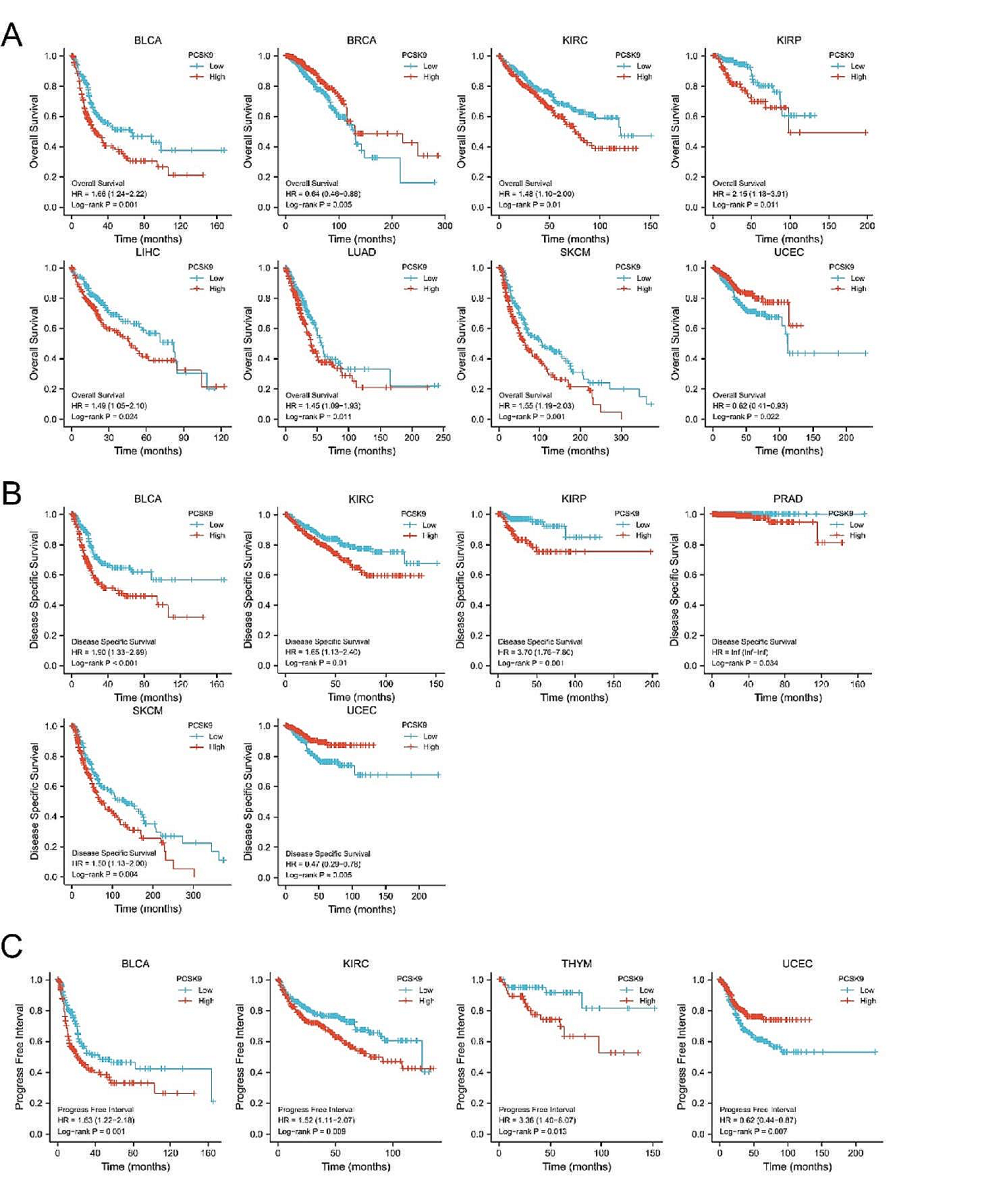
Correlation between PCSK9 gene expression and survival prognosis of cancers in TCGA. We used the survival package and survminer package to explore the correlation between PCSK9 expression and **(A)** overall survival, **(B)** disease-specific survival, and **(C)** progression-free interval across different tumors. The Kaplan-Meier curves with positive results are given (Log-rank test)

Given that PCSK9 expression was related to later stages, we next sought to identify its effect on disease prognosis. RNA-seq data and clinical data in HTSeq-FPKM format for multiple tumors were downloaded in TCGA. All tumor cases were divided into the PCSK9 high-expression group and the PCSK9 low-expression group according to the expression level with a cutoff of 50% of PCSK9 expression. Then, we analyzed the correlation between the expression levels of PCSK9 and patient prognosis across multiple tumor types. It showed that the high expression of PCSK9 was related to poorer OS in patients with BLCA (*P* = 0.001), KIRC (*P* = 0.01), KIRP (*P* = 0.011), LIHC (*P* = 0.024), lung adenocarcinoma (LUAD) (*P* = 0.011) and skin cutaneous melanoma (SKCM) (*P* = 0.001). In contrast, in patients with BRCA (*P* = 0.005), the high expression of PCSK9 was associated with prolonged OS (Fig. 2A, Table S3). In addition, we demonstrated that BLCA (*P* < 0.001), KIRC (*P* = 0.01), KIRP (*P* = 0.001), prostate adenocarcinoma (PRAD) (*P* = 0.034), and SKCM (*P* = 0.004) patients with higher expression of PCSK9 have a shorter DSS (Fig. 2B, Table S3). According to the PFI analysis, high PCSK9 expression was correlated with poor prognoses in BLCA (*P* = 0.001), KIRC (*P* = 0.009), and thymoma (THYM) (*P* = 0.013) patients (Fig. 2C, Table S3). In contrast, PCSK9 expression was linked to improved OS, DSS, and PFI of UCEC (*P* < 0.05) patients (Fig. 2).

Then, we analyzed the predictive power of PCSK9 expression for OS and obtained ROC curves (Figure S2). We found that in predicting the prognosis of patients with COAD, ESCA, KIRP, and READ, the predictive power of the PCSK9 expression level has a higher predictive ability (AUC > 0.9) (Figure S2A) than that of CHOL, KIRC, LIHC, LUAD, HNSC, PRAD, STAD and UCEC (AUC = 0.7 $\sim$ 0.9) (Figure S2B). The above data indicated that the high expression of PCSK9 tends to be related to poor prognoses in most categories of tumor patients.

### Regulatory effect of PCSK9 expression on tumor immune microenvironment

PCSK9 has been reported to regulate T-cell activation and infiltration in cancer. Therefore, we analyzed the correlations between PCSK9 expression and immune score, stromal score, and ESTIMATE score (Table S4). In most tumor types, PCSK9 expression was negatively correlated with the immune, stomal, and ESTIMATE scores. PCSK9 expression was positively correlated with immune score, stomal score, and ESTIMATE score in BLCA (*P* < 0.001), BRCA (*P* < 0.001), pheochromocytoma and paraganglioma (PCPG) (*P* < 0.001) and THCA(*P* < 0.001). In addition, PCSK9 expression was positively correlated with only stomal score or ESTIMATE score in DLBC (*P* < 0.05), KIRC (*P* < 0.05), sarcoma (SARC) (*P* < 0.01), and uveal melanoma (UVM) (*P* < 0.05).

**Fig. 3:**
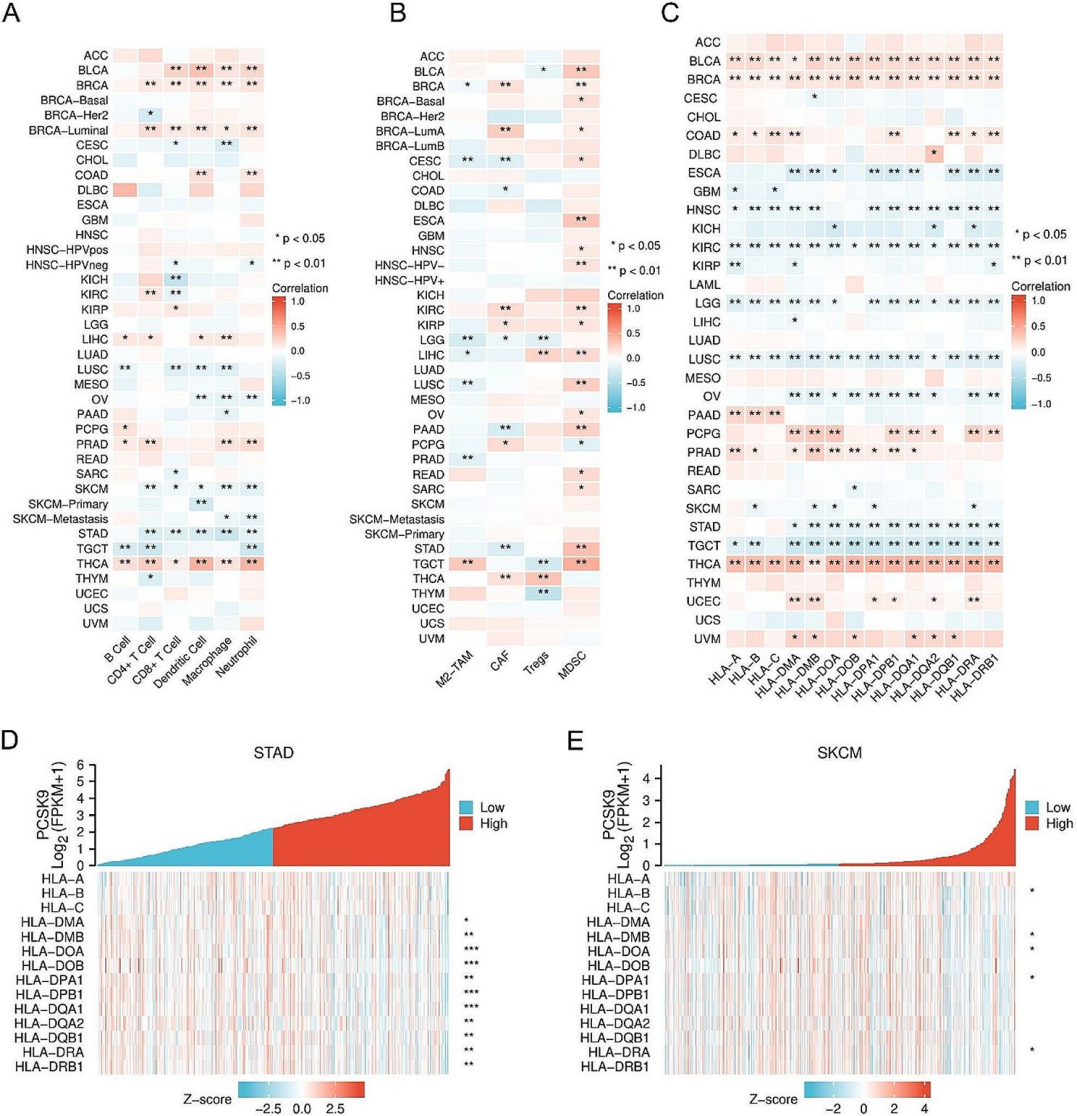
Regulatory effects of PCSK9 expression on tumor immune cells infiltration and MHC expression. **(A)** Heat map of correlation between PCSK9 expression and infiltration of B cell, CD8 + T cell, CD4 + T cell, macrophage, neutrophil, and DC. **(B)** Heat map of correlation between PCSK9 expression and infiltration of M2 macrophage, myeloid-derived suppressor cell, regulatory T cell, and cancer-associated fibroblast. **(C)** Heat map of correlation between PCSK9 expression and MHC molecules expression in pan-cancer. **(D)** Heat map of correlation between PCSK9 expression and MHC molecules expression in STAD. **(E)** Heat map of correlation between PCSK9 expression and MHC molecules expression in SKCM. *P < 0.05; **P < 0.01; ***P < 0.001)

Next, TIMER and TIMER2.0 were used to explore the potential relationships between different immune cell infiltration levels and PCSK9 gene expression in multiple tumors. Among the 39 TCGA tumor types and subtypes, the expression of PCSK9 in BRCA, BRCA-Luminal, and THCA was significantly positively correlated with the infiltration of B cells, CD8^+^ T cells, CD4^+^ T cells, macrophages, neutrophils, and DCs. However, the expression of PCSK9 was negatively correlated with the infiltration of five immune cell types except for B cells in SKCM (*P* < 0.05) and STAD (*P* < 0.01) (Fig. 3A). Moreover, the expression level of PCSK9 was negatively correlated with the infiltration of immunosuppressive cell types such as M2 macrophages, CAFs, and Tregs in brain lower grade glioma (LGG) (*P* < 0.05). Notably, PCSK9 expression was significantly positively correlated with MDSC infiltration in most tumors (Fig. 3B).

Moreover, we used the TIMER analysis website to analyze the correlation between the expression of PCSK9 and immune checkpoint molecules, including PDCD1, CD274, and CTLA4. The results showed that in BLCA (*P* < 0.001), BRCA (*P* < 0.001), COAD (*P* < 0.05), and THCA (*P* < 0.001), PCSK9 was positively correlated with PDCD1, CD274, and CTLA4 but negatively correlated in HNSC (*P* < 0.05) and testicular germ cell tumor (TGCT) (*P* < 0.01). (Table S5).

In addition, we analyzed the correlations between the expression levels of PCSK9 and MHC-I (human leukocyte antigen-A, B, C) and MHC-II (human leukocyte antigen-DMA, DMB, DOA, DOB, DPA1, DPB1, DQA1, DQA2, DQB1, DRA, DRB1). The results indicated that in BLCA (*P* < 0.05), BRCA (*P* < 0.01), and THCA (*P* < 0.01), PCSK9 expression was positively correlated with all subtypes of MHC-I and MHC-II, and the opposite trends were obtained in KIRC (*P* < 0.05) and LUSC (*P* < 0.05) (Fig. 3C). PCSK9 was negatively correlated with the expression of most MHC-II subtypes in STAD (*P* < 0.05) (Fig. 3D) and with several MHC-II subtypes in melanoma (Fig. 3E). Collectively, the above results suggested that PCSK9 has an immunosuppressive role in the main spectrum of cancer.

### PCSK9 inhibition upregulated MHC-II expression and mediated CD8^+^ T cell functional activation in vitro

**Fig. 4:**
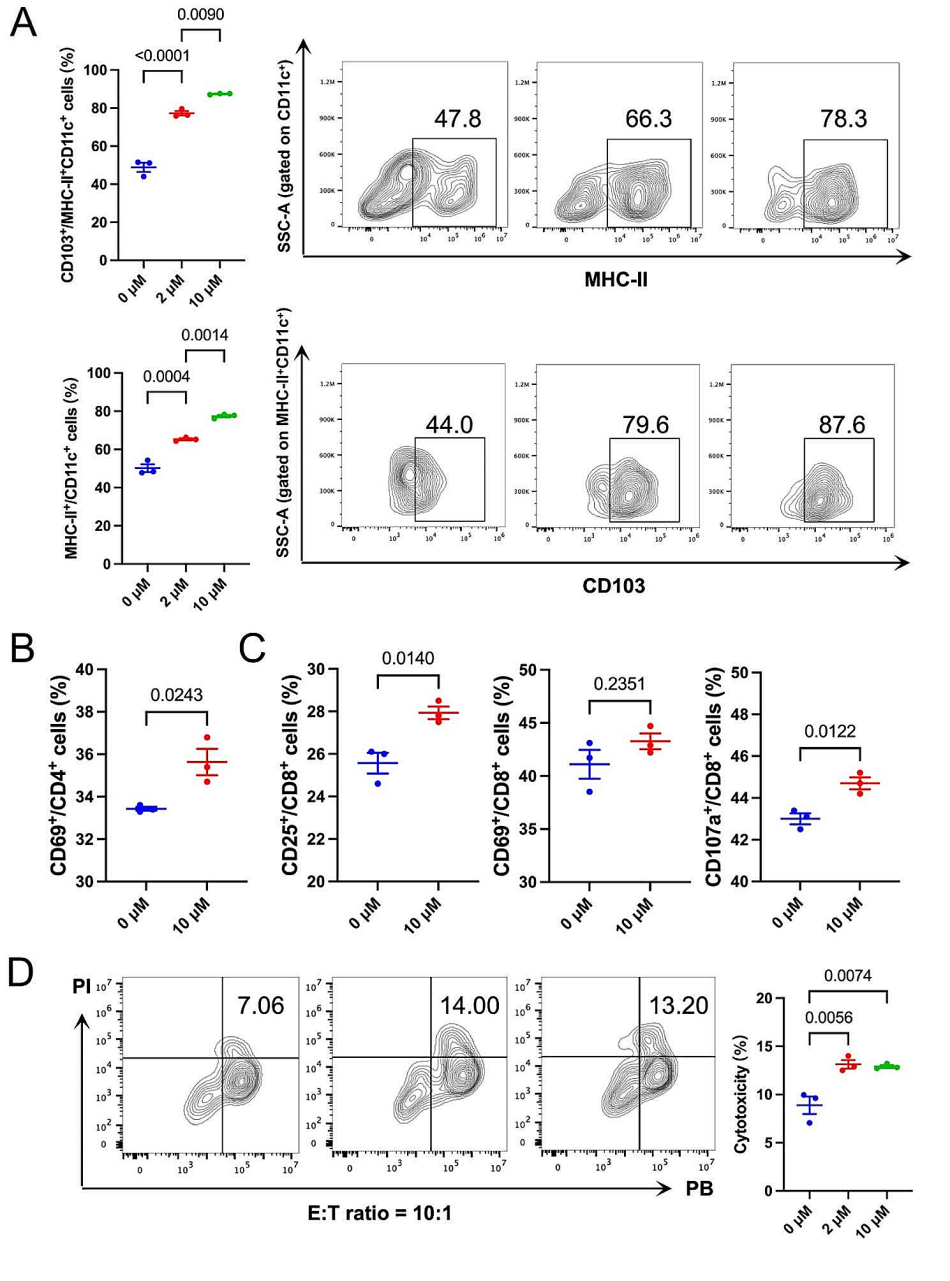
PCSK9 inhibition enhanced MHC-II expression and CD8^+^ T cell activation in vitro **(A)** Flow cytometry staining of the ratios of DCs (MHC-II^+^CD11c^+^) and CD103^+^ DCs in spleen cells after direct treatment of different concentrations (0, 2, and 10 µM) of PCSK9i for 48 h. **(B**-**C)** MFC tumor cells were pretreated with PCSK9i (0 and 10 µM) for 24 h and then cocultured with spleen cells for another 12 h. The activation marker expression on CD4^+^ T cells and CD8^+^ T cells was detected by flow cytometry. **(D)** Flow cytometry detection of the lysis of monolayer PB-labeled MFC tumor cells pretreated by different concentrations (0, 2, and 10 µM) of PCSK9i for 24 h and then co-cultured with T cells at an E: T ratio of 10:1. Data with error bars are shown as mean ± SEM

Given that PCSK9 was negatively correlated with MHC-II expression according to our bioinformatic analysis, especially in gastric cancer, we hypothesized that PCSK9 inhibition may upregulate MHC-II expression. Therefore, a PCSK9i (PCSK9 inhibitor), PF-06446846, was used to treat spleen cells extracted from 615-line mice, which was a feasible mouse variety for the syngeneic gastric cancer mouse model. MHC-II expression on DCs in spleen cells was dose-dependently enhanced (*P =* 0.0004; *P =* 0.0014), as well as the ratios of migrating DCs with antigen-presenting abilities (defined as CD103^+^) (*P <* 0.0001; *P =* 0.0090) (Fig. 4A). Since MHC-II could prime CD4^+^ T cell-based adaptive immunity and help activate CD8^+^ T cells, we further explored the impacts of PCSK9 inhibition on T cell functional status. Spleen cells were extracted from 615-line mice and then cocultured with PCSK9i-pretreated MFC gastric cancer cells. Results showed that CD4^+^ T cells were more activated in the PCSK9i-treated group, manifested as CD69 upregulation (*P =* 0.0243) (Fig. 4B). Additionally, CD8^+^ T cells obtained a strengthened activating (CD25^+^ and CD69^+^) (*P =* 0.0140; *P =* 0.2351) and cytotoxic (CD107a^+^) phenotype (*P =* 0.0122) (Fig. 4C). Moreover, the tumor-killing ability of T cells was dose-dependently improved via pretreatment of PCSK9i on tumor cells (*P =* 0.0056; *P =* 0.0074) (Fig. 4D). The above in vitro findings demonstrated the regulatory role of PCSK9 inhibition in upregulating MHC-II expression, which subsequently induced T-cell activation and function improvement.

### PCSK9 inhibition suppresses tumor growth and improves the TME via MHC-II upregulation in syngeneic gastric cancer mouse models

**Fig. 5:**
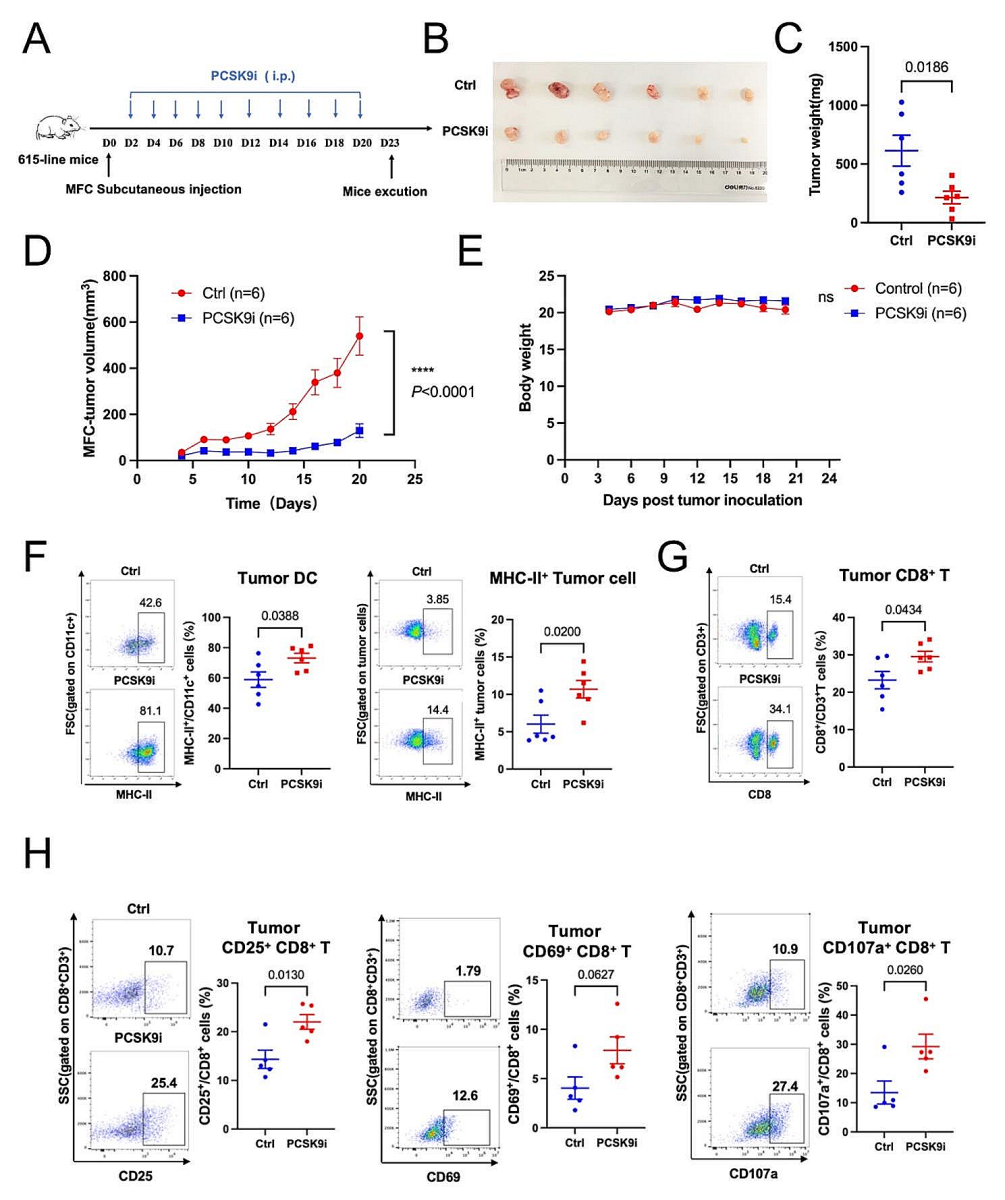
PCSK9 inhibition triggers anti-tumor effects in gastric cancer model **(A)** Schematic of PCSK9i treatment in MFC challenged syngeneic gastric cancer model. 615-line mice are s.c. injected with 10^6^ MFC cells (*n* = 6 per group) and treated i.p. with PCSK9i (5 mg/kg) or DMSO every other day for 18 days. Mice were sacrificed via cervical dislocation at the treatment endpoint, and **(B)** tumors were removed for analysis. **(C)** Tumor weight and **(D)** volumes of MFC-bearing 615-line mice treated with PCSK9i or DMSO (*n* = 6 per group). **(E)** Weights of 614-line mice in two groups. **(F**-**G)** The proportions of tumor-infiltrating MHC-II^+^/CD11c^+^ cells, MHC-II^+^ tumor cells, and CD8^+^/CD3^+^ cells were determined by flow cytometry. **(H)** The proportions of tumor-infiltrating CD25^+^/CD8^+^ cells, CD69^+^/CD8^+^ cells, and CD107a^+^/CD8^+^ cells were assessed by flow cytometry. Data with error bars are shown as mean ± SEM

As PCSK9 may facilitate the immunosuppressive TME in multiple cancers according to bioinformatic analysis, we aim to explore the immunoregulatory role of PCSK9 inhibition in vivo. First, we determined that PCSK9 protein was highly expressed in MFC and B16F10-OVA cell lines and could be successfully downregulated by PCSK9i (Figure S3). Then, we established a syngeneic gastric cancer mouse model by inoculating MFC cells subcutaneously (Fig. 5A). Results showed that tumor volumes in the PCSK9i-treated group were significantly reduced compared with the control group (*P* < 0.0001) (Fig. 5B, D). PCSK9i also led to remarkably lower tumor weights at the endpoint (*P* = 0.0186) (Fig. 5C). No significant change in body weight (Fig. 5E) or noticeable side effects on vital organs were observed (Figure S4A).

To further assess the regulatory impacts of PCSK9 inhibition on the TME, we prepared single-cell suspensions from the tumor tissues of gastric cancer-bearing mice at the experiment termination. Flow cytometry was employed to detect MHC-II expression on tumor-infiltrating DCs and tumor cells. We found that MHC-II expression on DCs and tumor cells increased after PCSK9 blockade (*P* = 0.0388; *P =* 0. 0.0200) (Fig. 5F). CD8^+^ T cell infiltration was enhanced, indicating the stimulation of anti-tumor immune responses (*P* = 0.0434) (Fig. 5G). To further investigate the activation and functional status of CD8^+^ T cells in vivo, we established another MFC mouse model as aforementioned and administered PCSK9i for five days. Flow cytometry measurements revealed that the proportions of CD25^+^ or CD69^+^ activated CD8^+^ T cells and CD107a^+^ cytotoxic subgroups were upregulated (*P* = 0.0130; *P =* 0.0627; *P =* 0.0260) (Fig. 5H), corroborating the improvement of T cell-based adaptive anti-tumor immunity. Moreover, CD25^+^ and CD69^+^/CD4^+^ T cells also modestly increased, suggesting the immune stimulation caused by MHC-II-mediated antigen presentation (Figure S5) (*P =* 0.0500; *P =* 0.2026). The gating strategy of the flow cytometry data is shown in the supplementary figure (Figure S6A, C, D, E). These findings indicate that PCSK9 inhibition could upregulate MHC-II expression on DCs and tumor cells in the TME of the mouse syngeneic model, leading to enhanced T cell-mediated tumor control.

### Combinatorial PCSK9 inhibition improved the anti-tumor effects of OVA-II peptide vaccines

**Fig. 6:**
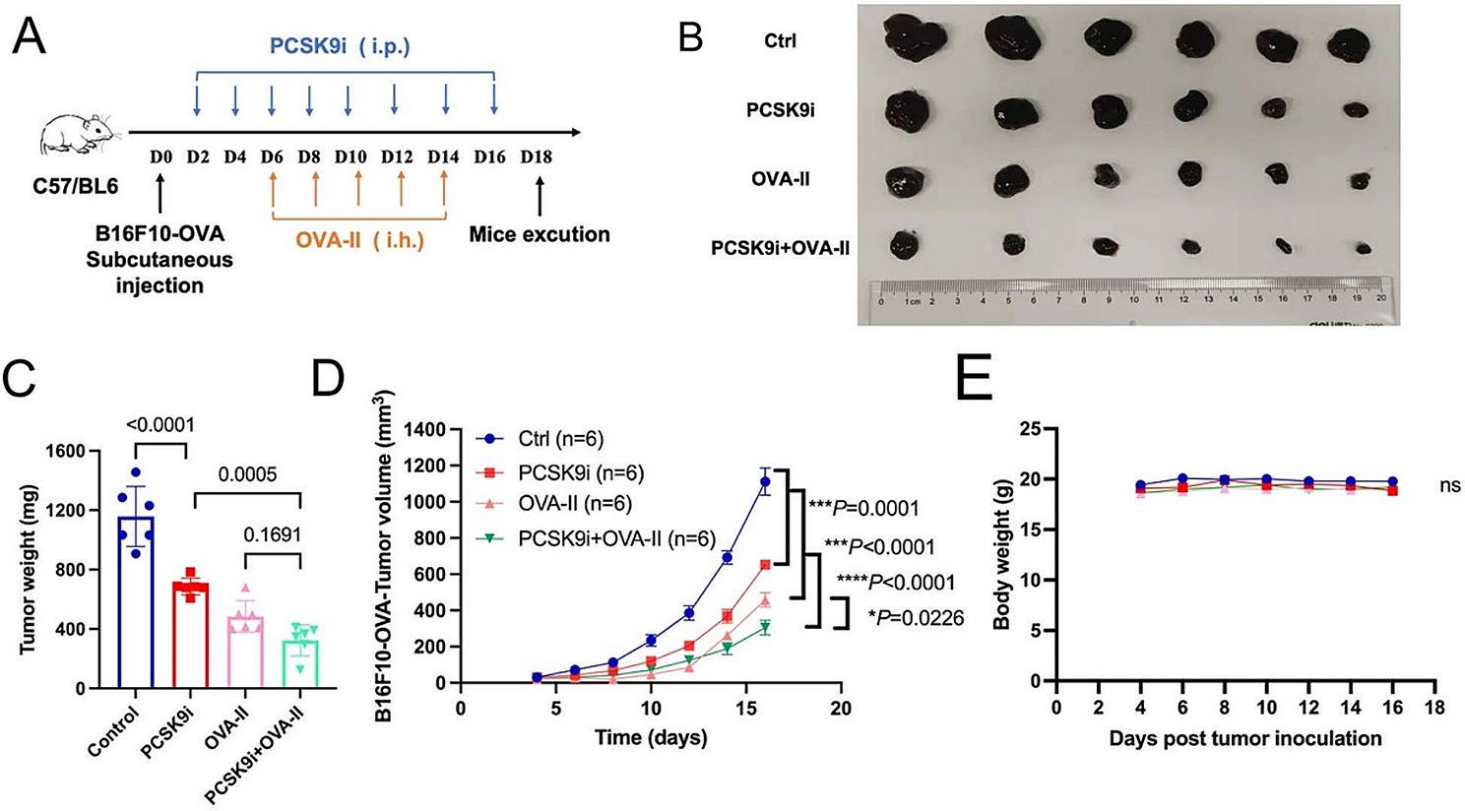
Combining PCSK9 inhibitors with OVA-II vaccines triggers better anti-tumor effects in the melanoma mouse model. **(A)** Schematic of PCSK9i combined with OVA-II vaccine treatment schedule in B16F10-OVA challenged syngeneic melanoma model. C57BL/6-line mice are s.c. injected with 10^5^ B16F10-OVA cells (*n* = 6 per group) and treated i.p. with PCSK9i (5 mg/kg), DMSO, treated s.c. with OVA-II vaccine (10ug/mouse), PCSK9i(5 mg/kg) combined with OVA-II vaccine (10ug/mouse) every other day for 18 days. Mice were sacrificed via cervical dislocation at the treatment endpoint, and **(B)** tumors were harvested for analysis. **(C**-**D)** Tumor weights and volumes of B16F10-OVA-bearing C57BL/6-line mice (*n* = 6 per group). **(E)** Weights of B16F10-OVA-bearing mice in two groups. Data with error bars are shown as mean ± SEM

Considering MHC-II mediates CD4^+^ T-cell epitope-initiated immune responses, we hypothesized that combining PCSK9 inhibitors with long peptide vaccines may achieve synergistic anti-tumor effects. A subcutaneous tumor model of B16F10-OVA melanoma was established and treated by the PCSK9 inhibitor and OVA-II peptide vaccines (Fig. 6A). Results showed that in addition to the remarkable anti-tumor effects of the PCSK9 inhibitor (*P* = 0.0001) or the OVA long peptide vaccine (*P* < 0.0001) alone, synergistic tumor-repressing effects were achieved by the combination of PCSK9 inhibitor with OVA-II vaccine than either PCSK9 inhibitor (*P* < 0.0001) or OVA-II peptide alone(*P* = 0.0226) (Fig. 6B, C, D). No apparent side effects were observed regarding tumor weights or important organs (Fig. 6E; Figure S4B). Furthermore, flow cytometry detection demonstrated that PCSK9 inhibition increased the infiltration ratio of CD8^+^ T cells (*P* = 0.0015) and DCs (*P* = 0.0024) and promoted the expression of MHC-II (*P* = 0.0097) on the surface of tumor cells (Figure S7). The gating strategy of the flow cytometry data is shown in the supplementary figure (Figure S5B-D). Our results suggested that PCSK9 inhibition is a promising combination therapy for MHC class-II restricted tumor vaccines.

## Discussion

Recently, cancer immunotherapy, represented by immune checkpoint inhibitors, has played a crucial role in the treatment of various cancer types, given its successful tumor control either alone or in combination with radiotherapy or chemotherapy [19]. However, therapeutic efficacies are still limited to subpopulations of patients [20]. Therefore, further exploration is warranted to identify predictive biomarkers for efficacy or develop feasible combination treatment strategies.

PCSK9 protein has been reported to participate in cholesterol metabolism and can regulate the progression of many diseases, including atherosclerosis and several tumor types, such as gastric cancer, liver cancer, small bowel cancer, lung cancer, breast cancer, etc. [21, 22]. Several studies have shown that PCSK9 can serve as an independent prognostic factor for multiple tumors and that high expression of PCSK9 can promote tumor progression through various mechanisms. However, through a literature search, we failed to retrieve any publications with a pan-cancer analysis of PCSK9 from the perspective of overall tumors. Thus, we comprehensively examined the expression of PCSK9 in 33 different tumors based on TCGA data to investigate the correlation between PCSK9 and cancer progression. Furthermore, we evaluated the modulation of PCSK9 on anti-tumor immunity and explored the value of targeting PCSK9 in immunotherapy.

Firstly, we found that PCSK9 expression was aberrantly elevated in different tumors and that high PCSK9 expression was associated with poor prognosis in most tumor types, although there are exceptions, such as BRCA and UCEC. OS, DSS, and PFI analyses of BLCA and KIRC suggested that patients with high PCSK9 expression have a poor prognosis, and high PCSK9 expression in KIRC is correlated with a worse physiological stage. The opposite result was obtained in UCEC. In addition, the role of PCSK9 in LIHC is controversial. He et al. reported that PCSK9 expression was lower in LIHC tissues than in normal tissues and inhibited HCC cell proliferation, cell cycle progression, and apoptosis by interacting with GSTP1 and suppressing JNK signaling [7]. However, Zhang et al. reported that high PCSK9 expression levels in hepatocellular carcinoma are an indicator of poor prognosis, and FASN-mediated anti-apoptotic effects play an important role in PCSK9‑induced hepatocellular carcinoma progression [6]. Our findings displayed that the mRNA expression of PCSK9 was higher in tumor tissues than in normal tissues in LIHC and correlated with poorer OS. Notably, previous studies have shown that PCSK9 mRNA and protein levels are significantly elevated in gastric cancer tissues and are related to tumor progression and poor survival [5]. Our study’s expression results are consistent with those of published studies [5, 10].

Immune score and stromal score have been documented to predict cancer prognosis []. Tumor-infiltrating immune cells and cancer-associated fibroblasts, which are key components of the TME, play a determinative role in tumor progression [23]. The recruitment and differentiation of immunosuppressive cells such as M2 macrophages, MDSCs, Tregs, and CAFs can induce immune tolerance [24]. In addition, tumors could upregulate immune checkpoints to avoid being detected and killed by the host immune system [25]. Here, we explored the correlations of PCSK9 with anti-tumor immunity, including the immune score, immune cell infiltration, and the relationship with the expression level of immune checkpoints. We found that PCSK9 mediates the positive immune microenvironment in BRCA and THCA; however, the suppressive immune microenvironment in both STAD and TGCT, suggesting that blocking PCSK9 could benefit some cancer types. In TGCTs, PCSK9 is associated with the downregulation of immune checkpoint expression. In BLCA, BRCA, THCA, and UVM, PCSK9 is associated with high expression of immune checkpoints, indicating immune tolerance and potential benefits from immune checkpoint inhibitor therapy. However, more clinical data are needed to confirm these hypotheses.

Previous studies have shown that PCSK9 mediates immune escape and immunotherapy resistance by hampering the infiltration and function of CD8^+^ T cells and causing T cell dysfunction [16, 17, 26]. Moreover, PCSK9 was found to have regulatory effects on DCs in non-tumor diseases [27]. These findings supported PCSK9 as an immune regulator. In previous reports, Liu et al. discovered that PCSK9 could disrupt the recycling of MHC-I to the cell surface by promoting its relocation and degradation in the lysosome through physical association. Inhibiting PCSK9 caused a significant increase in tumor cell surface MHC-I expression, which promoted robust intra-tumoral infiltration of cytotoxic T-cells [16]. Different from MHC-I, MHC-II molecules are mainly expressed by antigen-presenting cells such as dendritic cells, macrophages, and B cells, and present exogenous antigens to CD4^+^T cells. They can also be expressed by tumor cells [28]. Related studies have shown that tumor-specific MHC-II molecules are related to better prognoses of cancer patients and tumor resistance in mouse models [29–33]. Interestingly, our bioinformatics analysis has verified the negative relations of PCSK9 on MHC-II expression, especially in the gastric cancer and melanoma cohorts. Then, we revealed that PCSK9i could elevate MHC-II^+^ and antigen-presenting CD103^+^ DC proportions in spleen cells, sensitize tumor cells to be killed by T cells, and indirectly activate both CD4^+^ and CD8^+^ T cells in vitro. To further explore the role of PCSK9 in the TME, we established the MFC-bearing 615 mouse model and illustrated the effective tumor suppression of PCSK9i. For the first time, we demonstrated that PCSK9 inhibition could promote MHC-II expression on both DCs and tumor cells in the TME, corroborating the regulatory functions of PCSK9 on MHC-II expression. We also found that CD4^+^ T cell activation and the infiltrating levels and functional status of CD8^+^ T cells were improved. Given that the MHC-II restricted peptide cancer vaccine relies on MHC-II molecules to prime initial antigen-specific immune responses, we further combine PCSK9 inhibitors with OVA-II tool peptide vaccine in treating syngeneic B16-OVA melanoma model and proved that combinatorial treatment can achieve better anti-tumor effects than monotherapy.

Previous studies demonstrated that Ras–MAPK activity can suppress the expression of MHC-I and MHC-II induced by IFNγ, and tumor cells can circumvent antigen presentation pathways by activating the MAPK pathway [34]. Furthermore, Xu et al. found that PCSK9 promotes gastric cancer metastasis and suppresses apoptosis by facilitating the MAPK signaling pathway [5]. Therefore, we hypothesize that PCSK9 inhibits the expression level of MHC-II on the surface of tumor cells by activating the MAPK signaling pathway. However, further investigation is needed to clarify the underlying mechanisms. Additionally, a limitation of our study was that we did not identify tumor cells in a more specific manner, such as sorting or staining EpCAM^+^ cells, which could be improved in future studies.

## Conclusion

Taken together, our pan-cancer analysis showed that the expression level of PCSK9 was significantly correlated with clinical characteristics, prognosis, immune matrix score, immune cell infiltration, immune checkpoint expression, and major histocompatibility complex expression in patients with multiple tumors, which is helpful for understanding the role of PCSK9 in tumorigenesis in various cancers from the perspective of clinical data. In addition, we explored the immune regulatory effect of PCSK9 inhibition both in vitro and in vivo. In syngeneic mouse models of gastric cancer, PCSK9 inhibition was demonstrated to upregulate DC infiltration and tumor cell MHC-II expression and subsequently enhance CD8^+^ T cell activation to realize potent tumor repression. Furthermore, combining PCSK9 inhibitors with OVA-II peptide vaccines can achieve better anti-tumor effects, providing evidence for applying PCSK9 inhibition as a promising immunoregulatory therapeutic strategy for cancer treatment.

### Electronic supplementary material

Below is the link to the electronic supplementary material.

Supplementary Material 1

## Data Availability

Bioinformatics analysis data are available in the public, open access repository: The Cancer Genome Atlas (TCGA) (https://cancergenome.nih.gov/), other data supporting the findings of this study are available within the article or its supplementary materials.

## Abbreviations

BLCA: bladder urothelial carcinoma
BRCA: breast invasive carcinoma
CAFs: cancer-associated fibroblasts
CESC: cervical squamous cell carcinoma
CHOL: cholangiocarcinoma
COAD: colon adenocarcinoma
CTLs: cytotoxic T lymphocytes
DCs: dendritic cells
DLBC: diffuse large B-cell lymphoma
DSS: disease-specific survival
ESCA: esophageal carcinoma
HNSC: head and neck squamous cell carcinoma
IFN-γ: interferon-γ
IL-17: interleukin-17
KIRC: kidney renal clear cell carcinoma
LDL: low-density lipoprotein
LDLR: low-density lipoprotein receptor
LGG: brain lower grade glioma
LIHC: liver hepatocellular carcinoma
LUAD: lung adenocarcinoma
LUSC: lung squamous cell carcinoma
MAPK: mitogen-activated protein kinase
MDSCs: myeloid-derived suppressor cells
MESO: mesothelioma
MHC-I: major histocompatibility complex-I
MHC-II: major histocompatibility complex-II
OS: overall survival
PCPG: pheochromocytoma and paraganglioma
PCSK9: Proprotein convertase subtilisin/kexin type 9
PFI: progression-free interval
PRAD: prostate adenocarcinoma
READ: rectum adenocarcinoma
ROCs: receiver operating characteristic curves
SARC: sarcoma
SKCM: skin cutaneous melanoma
STAD: stomach adenocarcinoma
TCGA: The Cancer Genome Atlas
TGCT: testicular germ cell tumors
THCA: thyroid carcinoma
THYM: thymoma
TME: tumor microenvironment
TNF-α: tumor necrosis factor-α
Tregs: regulatory T cells
UCEC: uterine corpus endometrial carcinoma
UVM: uveal melanoma
