# Supplementary material for: Targeting PCSK9 to upregulate MHC-II on the surface of tumor cells in tumor immunotherapy
Source: BMC Cancer. 2024 Apr 10;24:445. doi: 10.1186/s12885-024-12148-2 (PMC11007992; doi:10.1186/s12885-024-12148-2)
Supplement: Supplementary file 1 — Supplementary Material 1 [file 12885_2024_12148_MOESM1_ESM.docx]

**Supporting Information**

7 supporting figures, 5 supporting tables

**Figure S1.** **PCSK9 expression and its correlation with clinical characteristics.** **(A)** Expression level of PCSK9 in paired samples in CHOL and LUSC. **(B)** Correlation between PCSK9 expression and T stage in different tumor types. **(C)** Correlation between PCSK9 expression and N stage in different tumor types. **(D)** Correlation between PCSK9 expression and pathological or clinical stage in different tumor types. **P* < 0.05; ***P* < 0.01; ****P* < 0.001.

**Figure S2. ROC curves of PCSK9 expression levels in different cancers**. **(A)** PCSK9 expression has a high predictive ability in predicting the prognosis of COAD, ESCA, KIRP and READ. **(B)** In predicting the prognosis of CHOL, KIRC, LIHC, LUAD, HNSC, PRAD, STAD, UCEC, the predictive ability of PCSK9 expression level has certain accuracy.

**Figure S3. Western blot analysis of PCSK9 expression in MFC and B16F10-OVA cells before and after PCSK9i treatment. (A)** PCSK9 expression levels in MFC and B16F10-OVA cells after PCSK9i treatment was tested by Western Blot. Blots were cropped to fit the specific range of protein qualities and the cprresponding unprocessed full blots were displayed in **(B)**. The concentration of PCSK9 inhibitor was 10 μM.

**Figure S4. Safety evaluation of PCSK9i treatment in mouse organs was shown by hematoxylin and eosin (H&E) staining (Scale bars, 2.5 µm).** **(A)** Mouse organs removed from MFC challenged syngeneic gastric cancer model. **(B)** Mouse organs removed from B16F10-OVA challenged syngeneic melanoma model.

**Figure S5. Enhanced CD4^+^ T cell activation by PCSK9i in the gastric cancer mouse model**

The ratios of CD25^+^CD4^+^ T cells and CD69^+^CD4^+^ T cells were measured by flow cytometry. Data with error bars are shown as mean ± SEM.

**Figure S6. The gating strategy of the flow cytometry data.** (A) Flow cytometry gating strategy to quantitate the numbers of CD8^+^ T cells in gastric cancer murine tumors. (B) Flow cytometry gating strategy to quantitate the numbers of CD8^+^ T cells in melanoma murine tumors. (C) Flow cytometry gating strategy to quantitate the numbers of DC in both gastric cancer and melanoma murine tumors. (D) Flow cytometry gating strategy to quantitate the numbers of MHC-II^+^ tumor cells in both gastric cancer and melanoma murine tumors. (E) Flow cytometry gating strategy to quantitate the ratios of CD25^+^, CD69^+^, and CD107a^+^ in CD8^+^ or CD4^+^ T cells in gastric cancer murine tumors.

**Figure S7.** The proportions of CD8^+^ / CD3^+^ cells, MHC-II^+^/ CD11c^+^ cells, MHC-II^+^ cells in tumors removed in gastric cancer model were determined by flow cytometry. Data with error bars are shown as mean ± SEM.

**Table S1 The difference of PCSK9 expression between tumor and normal samples.**

**Table S2 Correlation between PCSK9 expression and TNM stage and pathological or clinical stage in different cancers.**

**Table S3 Survival prognostic analysis data of PCSK9 expression in different cancers.**

**Table S4 Correlation between PCSK9 expression and ImmuneScore, StromalScore, and ESTIMATEScore in different cancers.**

**Table S5 Correlation between PCSK9 expression and PDCD1 expression, CD274 expression and CTLA4 expression in different cancers.**

**Figure S1**

**
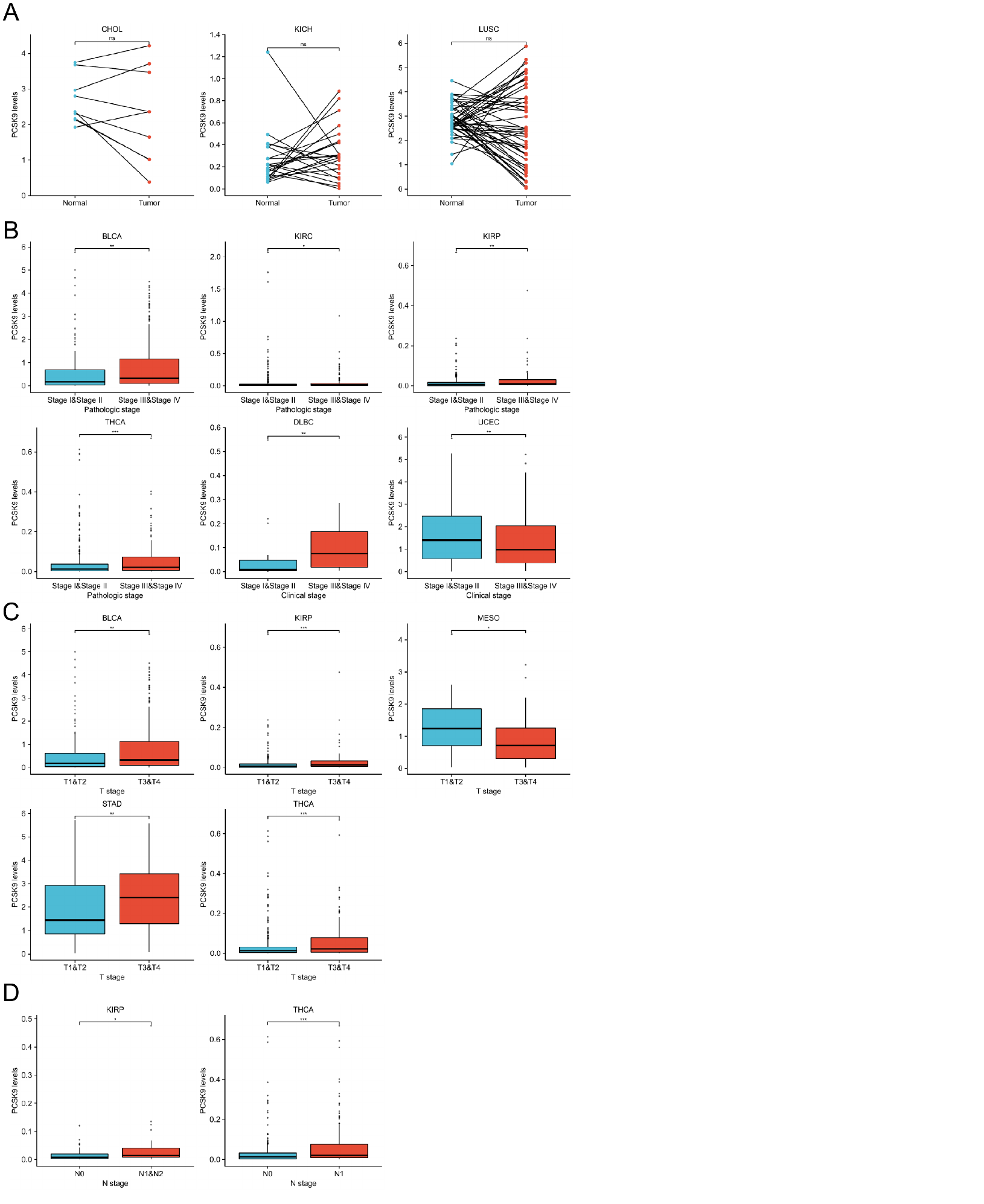
**

**Figure S2**

**
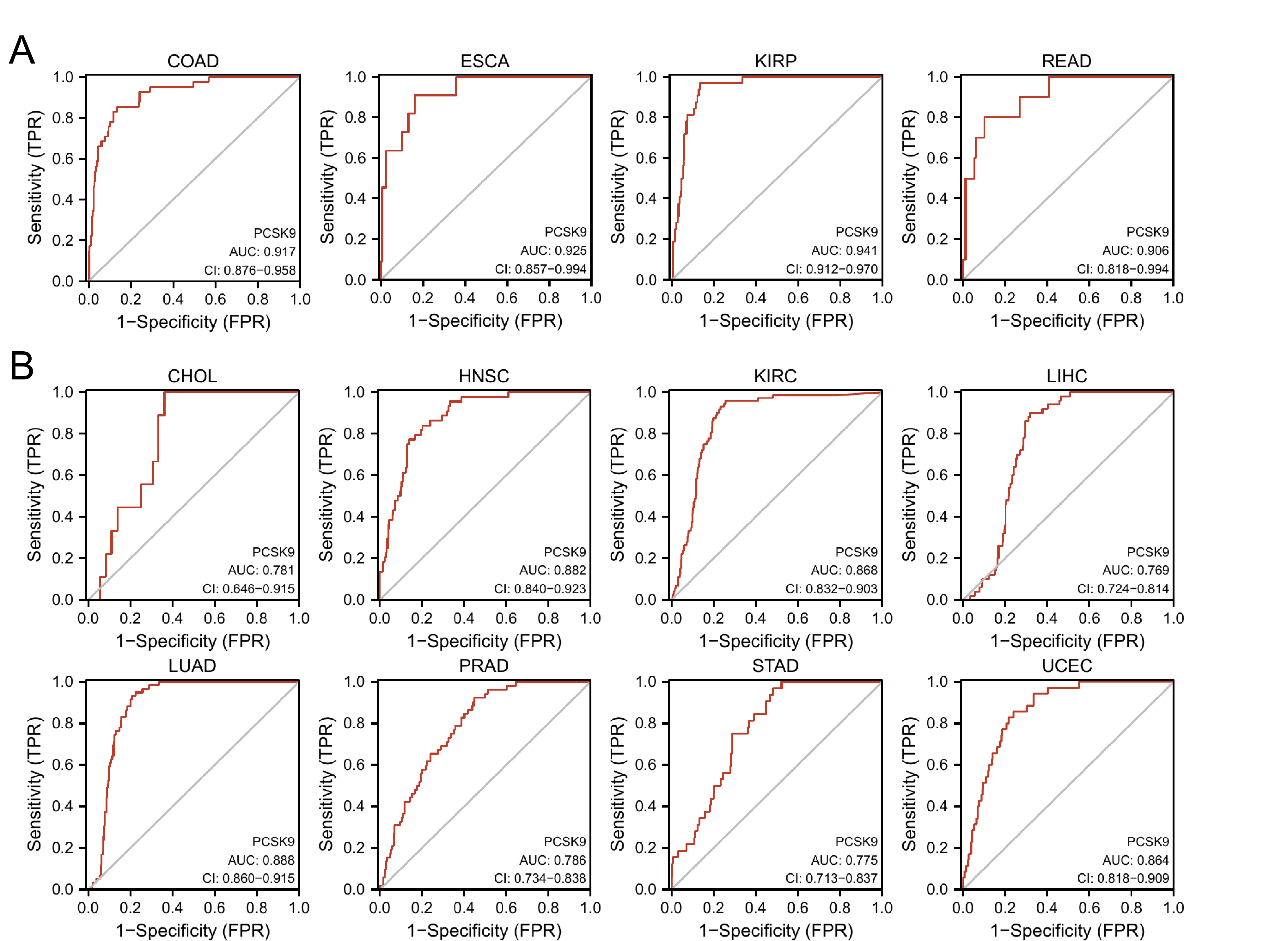
**

**Figure S3**


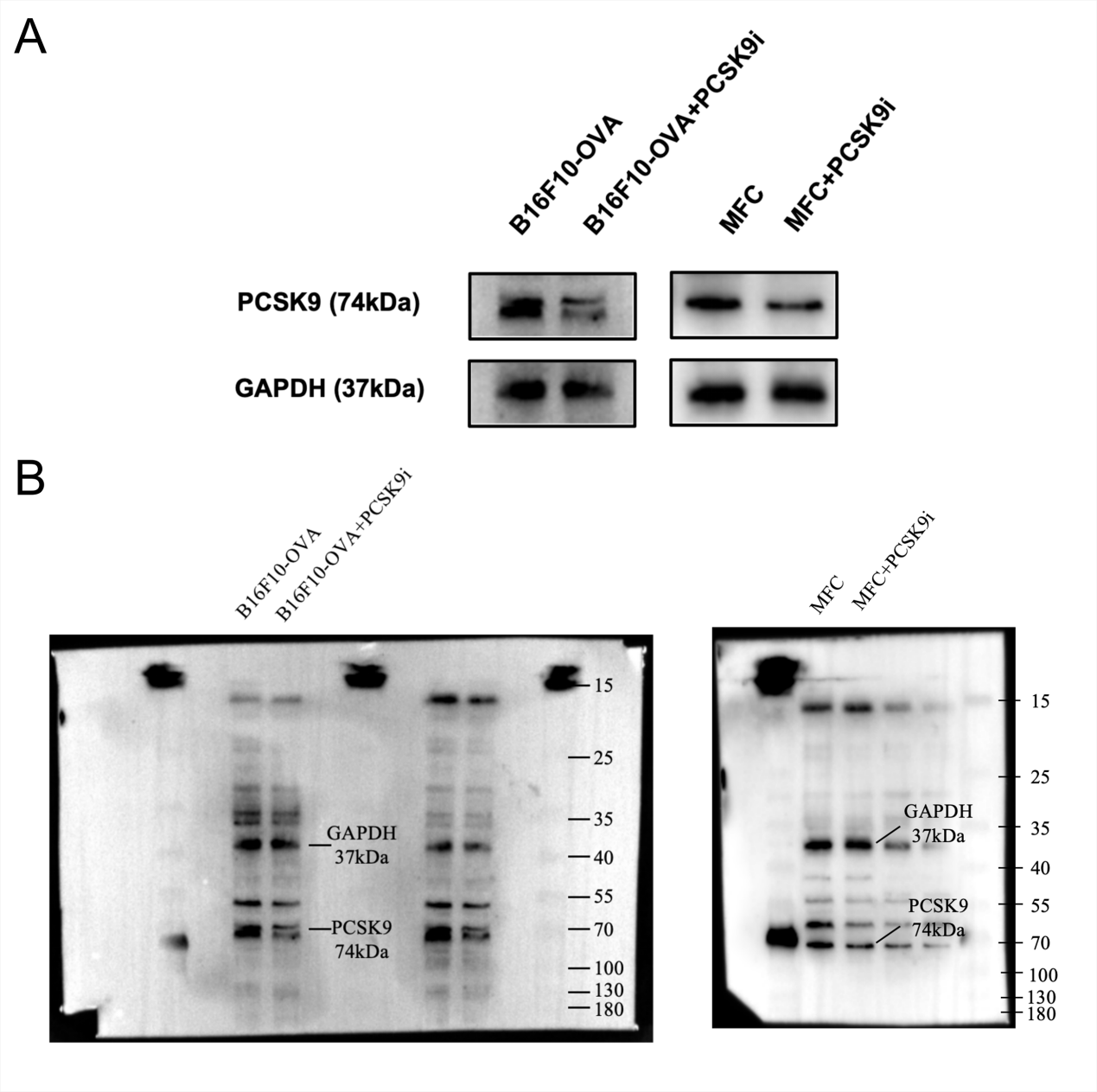


**Figure S4**

**
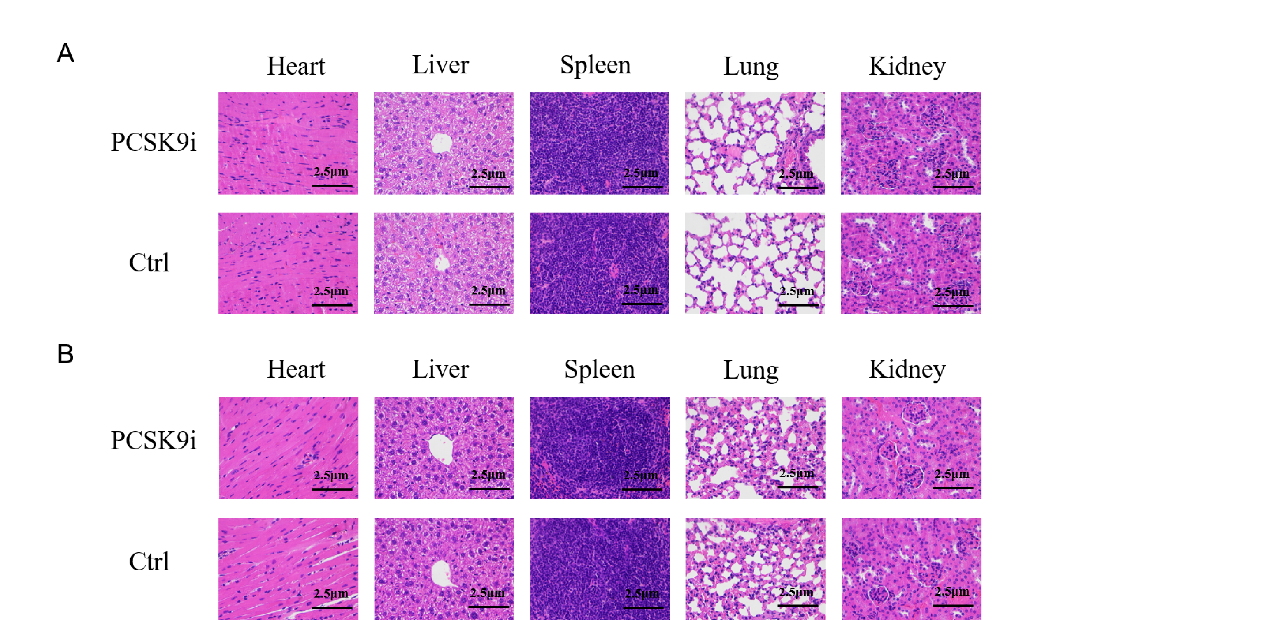
**

**Figure S5**

**
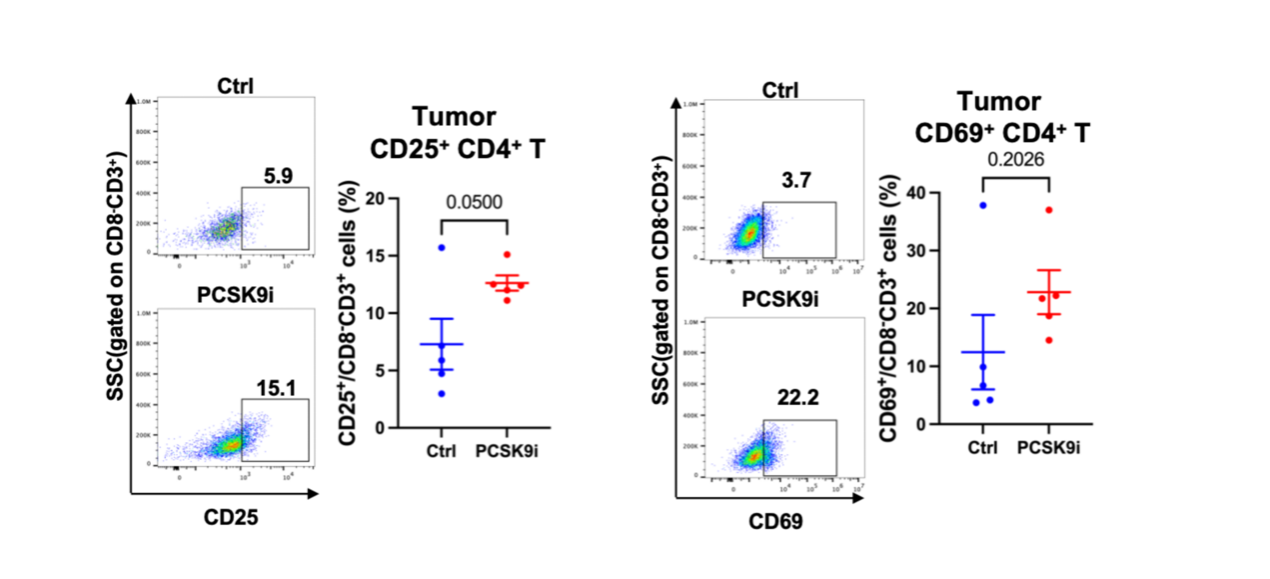
**

**Figure S6**

**
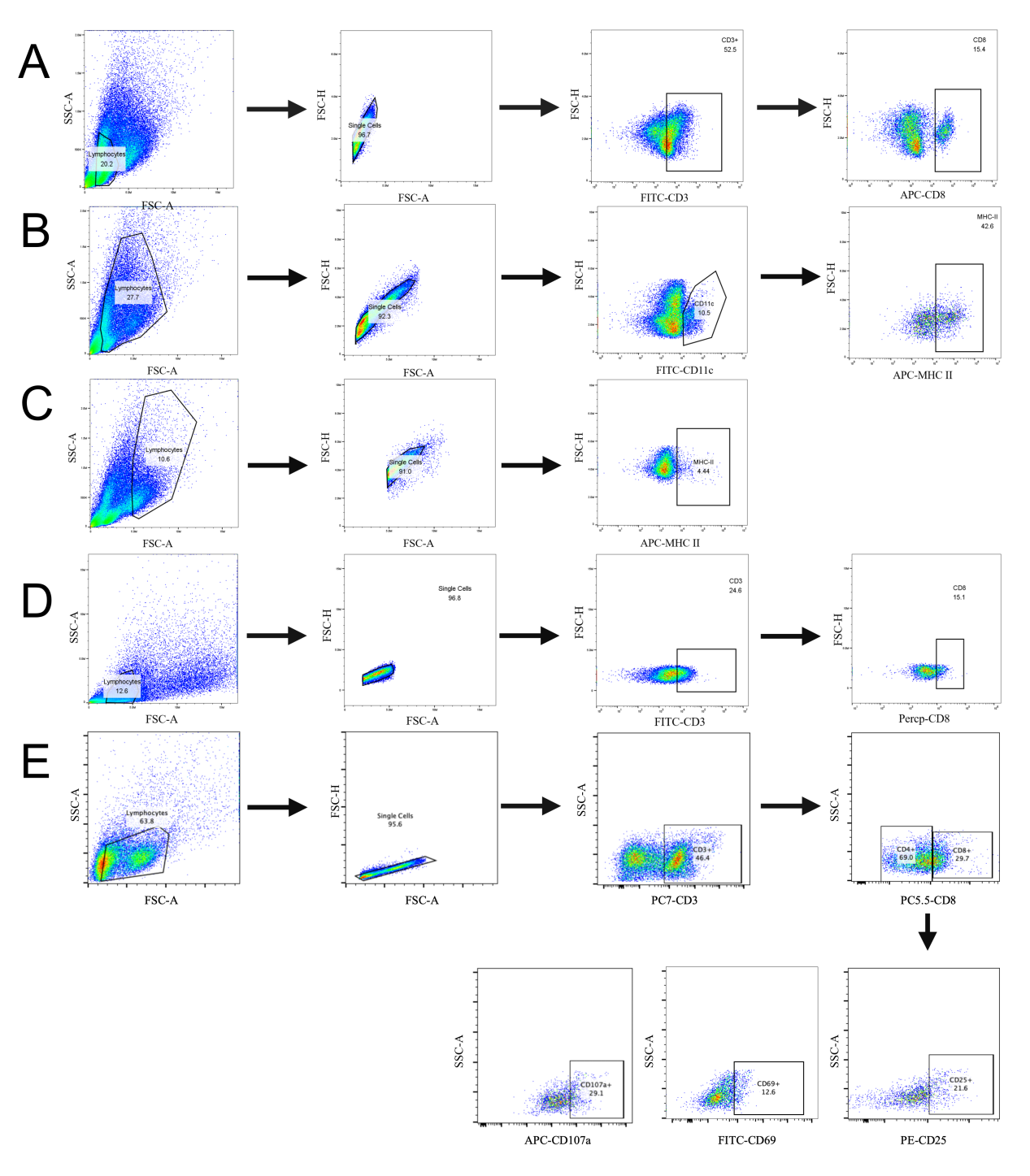
**

**Figure S7**

**
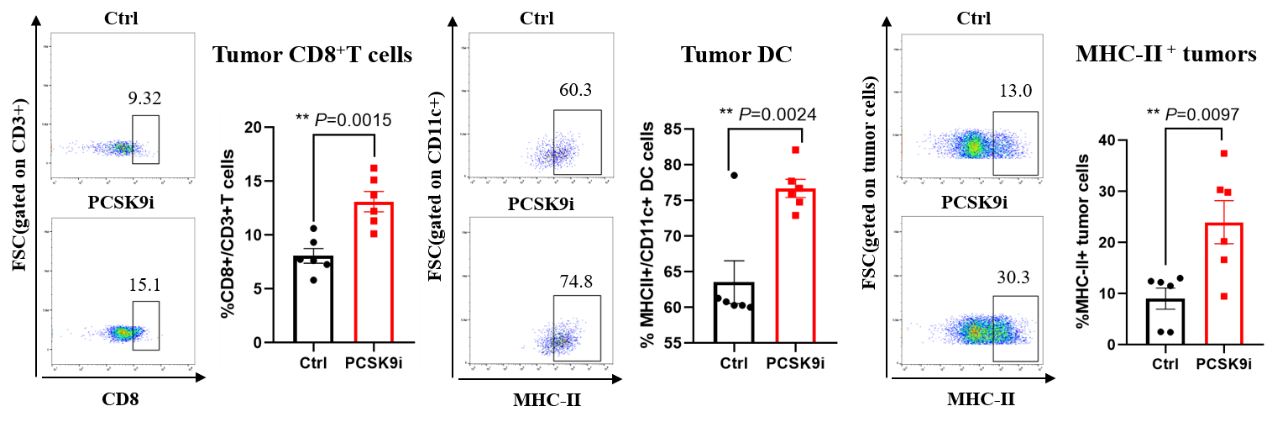
**

| **Table S1 The difference of PCSK9 expression between tumor and normal samples.** | | | | | | | | |
| --- | --- | --- | --- | --- | --- | --- | --- | --- |
| **Cancer types** | **Unpaired samples** | | **PCSK9 expression** | ***P-*value** | **Paired samples** | | **PCSK9 expression** | ***P-*value** |
|  | **normal(n)** | **tumor(n)** |  |  | **normal(n)** | **tumor(n)** |  |  |
| ACC | 0 | 79 | —— | —— | —— | —— | —— | —— |
| BLCA | 19 | 414 | ns | ns | 19 | 19 | up | * |
| BRCA | 113 | 1109 | up | *** | 113 | 113 | up | *** |
| CESC | 3 | 306 | up | * | —— | —— | —— | —— |
| CHOL | 9 | 36 | down | * | 9 | 9 | ns | ns |
| COAD | 41 | 480 | up | *** | 41 | 41 | up | *** |
| DLBC | 0 | 48 | —— | —— | —— | —— | —— | —— |
| ESCA | 11 | 162 | up | *** | 11 | 11 | up | *** |
| GBM | 5 | 169 | ns | ns | —— | —— | —— | —— |
| HNSC | 44 | 502 | up | *** | 44 | 44 | up | *** |
| KICH | 24 | 65 | ns | ns | 24 | 24 | ns | ns |
| KIRC | 72 | 539 | down | *** | 72 | 72 | down | *** |
| KIRP | 32 | 289 | down | *** | 32 | 32 | down | *** |
| LAML | 0 | 151 | —— | —— | —— | —— | —— | —— |
| LGG | 0 | 529 | —— | —— | —— | —— | —— | —— |
| LIHC | 50 | 374 | up | *** | 50 | 50 | up | *** |
| LUAD | 59 | 535 | down | *** | 59 | 59 | down | *** |
| LUSC | 49 | 502 | down | ** | 49 | 49 | ns | ns |
| MESO | 0 | 86 | —— | —— | —— | —— | —— | —— |
| OV | 0 | 379 | —— | —— | —— | —— | —— | —— |
| PAAD | 4 | 178 | ns | ns | —— | —— | —— | —— |
| PCPG | 3 | 183 | down | * | —— | —— | —— | —— |
| PRAD | 52 | 499 | down | *** | 52 | 52 | down | *** |
| READ | 10 | 167 | up | *** | 10 | 10 | up | ** |
| SARC | 2 | 263 | —— | —— | —— | —— | —— | —— |
| SKCM | 1 | 471 | —— | —— | —— | —— | —— | —— |
| STAD | 32 | 375 | up | *** | 32 | 32 | up | *** |
| TGCT | 0 | 156 | —— | —— | —— | —— | —— | —— |
| THCA | 58 | 510 | up | *** | 58 | 58 | up | *** |
| THYM | 2 | 119 | —— | —— | —— | —— | —— | —— |
| UCEC | 35 | 552 | up | *** | 35 | 35 | up | *** |
| UCS | 0 | 56 | —— | —— | —— | —— | —— | —— |
| UVM | 0 | 80 | —— | —— | —— | —— | —— | —— |
| ns *P ≧* 0.05; **P* < 0.05; ***P* < 0.01; ****P* < 0.001. | | | | | | | | |

| **Table S2 Correlation between PCSK9 expression and TNM stage and pathological or clinical stage in different cancers.** | | | | |
| --- | --- | --- | --- | --- |
| **Cancer types** | **Pathologic stage or clinical stage (Stage III&Stage IV vs. Stage I&Stage II)** | **T stage (T3&T4 vs. T1&T2)** | **N stage (N1&N2&N3 vs. N0)** | **M stage (M1 vs. M0)** |
| ACC | ns | ns | ns | ns |
| BLCA | ** | ** | ns | ns |
| BRCA | ns | ns | ns | ns |
| CESC | ns | ns | ns | ns |
| CHOL | ns | ns | ns | ns |
| COAD | ns | ns | ns | ns |
| DLBC | ** | —— | —— | —— |
| ESCA | ns | ns | ns | ns |
| GBM | —— | —— | —— | —— |
| HNSC | ns | ns | ns | ns |
| KICH | ns | ns | ns | —— |
| KIRC | * | ns | ns | ns |
| KIRP | ** | *** | * | ns |
| LAML | —— | —— | —— | —— |
| LGG | —— | —— | —— | —— |
| LIHC | ns | ns | ns | ns |
| LUAD | ns | ns | ns | ns |
| LUSC | ns | ns | ns | ns |
| MESO | ns | * | ns | ns |
| OV | —— | —— | —— | —— |
| PAAD | ns | ns | ns | ns |
| PCPG | —— | —— | —— | —— |
| PRAD | —— | ns | ns | ns |
| READ | ns | ns | ns | ns |
| SARC | —— | —— | —— | —— |
| SKCM | ns | ns | ns | ns |
| STAD | ns | ** | ns | ns |
| TGCT | ns | ns | ns | ns |
| THCA | *** | *** | *** | ns |
| THYM | —— | —— | —— | —— |
| UCEC | ** | —— | —— | —— |
| UCS | ns | —— | —— | —— |
| UVM | ns | ns | ns | ns |
| ns *P ≧* 0.05; **P* < 0.05; ***P* < 0.01; ****P* < 0.001. | | | | |

| **Table S3 Survival prognostic analysis data of PCSK9 expression in different cancers.** | | | | | | |
| --- | --- | --- | --- | --- | --- | --- |
| **Cancer types** | **OS** | ***P*-value** | **DSS** | ***P*-value** | **PFI** | ***P*-value** |
| ACC | ns | 0.706 | ns | 0.561 | ns | 0.311 |
| BLCA | unfavor | 0.001 | unfavor | <0.001 | unfavor | 0.001 |
| BRCA | favor | 0.005 | ns | 0.114 | ns | 0.341 |
| CESC | ns | 0.288 | ns | 0.541 | ns | 0.781 |
| CHOL | ns | 0.676 | ns | 0.339 | ns | 0.482 |
| COAD | ns | 0.393 | ns | 0.352 | ns | 0.312 |
| DLBC | ns | 0.269 | ns | 0.208 | ns | 0.457 |
| ESCA | ns | 0.85 | ns | 0.932 | ns | 0.707 |
| GBM | ns | 0.324 | ns | 0.257 | ns | 0.151 |
| HNSC | ns | 0.747 | ns | 0.684 | ns | 0.359 |
| KICH | ns | 0.306 | ns | 0.263 | ns | 0.104 |
| KIRC | unfavor | 0.01 | unfavor | 0.01 | unfavor | 0.009 |
| KIRP | unfavor | 0.011 | unfavor | 0.001 | ns | 0.179 |
| LAML | ns | 0.914 | —— | —— | —— | —— |
| LGG | ns | 0.997 | ns | 0.928 | ns | 0.477 |
| LIHC | unfavor | 0.024 | ns | 0.059 | ns | 0.195 |
| LUAD | unfavor | 0.011 | ns | 0.158 | ns | 0.133 |
| LUSC | ns | 0.941 | ns | 0.089 | ns | 0.315 |
| MESO | ns | 0.207 | ns | 0.257 | ns | 0.286 |
| OV | ns | 0.133 | ns | 0.155 | ns | 0.321 |
| PAAD | ns | 0.095 | ns | 0.096 | ns | 0.07 |
| PCPG | ns | 0.488 | ns | 0.971 | ns | 0.369 |
| PRAD | ns | 0.103 | unfavor | 0.034 | ns | 0.817 |
| READ | ns | 0.154 | ns | 0.688 | ns | 0.854 |
| SARC | ns | 0.615 | ns | 0.999 | ns | 0.984 |
| SKCM | unfavor | 0.001 | unfavor | 0.004 | ns | 0.161 |
| STAD | ns | 0.499 | ns | 0.502 | ns | 0.677 |
| TGCT | ns | 0.45 | ns | 0.579 | ns | 0.297 |
| THCA | ns | 0.678 | —— | —— | ns | 0.895 |
| THYM | ns | 0.4 | ns | 0.066 | unfavor | 0.013 |
| UCEC | favor | 0.022 | favor | 0.005 | favor | 0.007 |
| UCS | ns | 0.673 | ns | 0.818 | ns | 0.828 |
| UVM | ns | 0.16 | ns | 0.186 | ns | 0.181 |
| ns, PCSK9 expression is not correlated with prognosis; favor, high expression of PCSK9 was related to poor prognosis; unfavor, low expression of PCSK9 was related to poor prognosis. | | | | | | |

| **Table S4 Correlation between PCSK9 expression and ImmuneScore, StromalScore, and ESTIMATEScore in different cancers.** | | | | | | |
| --- | --- | --- | --- | --- | --- | --- |
| **Type of Cancer** | **immune score** | | **stromal score** | | **ESTIMATE score** | |
|  | **R** | ***P*** | **R** | ***P*** | **R** | ***P*** |
| ACC | 0.11 | 0.332 | 0.17 | 0.131 | 0.15 | 0.184 |
| BLCA | 0.21 | <0.001 | 0.24 | <0.001 | 0.25 | <0.001 |
| BRCA | 0.17 | <0.001 | 0.33 | <0.001 | 0.26 | <0.001 |
| CESC | -0.1 | 0.077 | -0.17 | 0.002 | -0.14 | 0.013 |
| CHOL | -0.056 | 0.744 | -0.065 | 0.704 | -0.035 | 0.839 |
| COAD | 0.058 | 0.202 | 0.002 | 0.966 | 0.034 | 0.453 |
| DLBC | 0.28 | 0.056 | 0.33 | 0.023 | 0.39 | 0.006 |
| ESCA | -0.24 | 0.002 | -0.099 | 0.211 | -0.17 | 0.03 |
| GBM | -0.077 | 0.316 | 0.037 | 0.637 | -0.032 | 0.678 |
| HNSC | -0.24 | <0.001 | -0.14 | 0.001 | -0.22 | <0.001 |
| KICH | -0.11 | 0.391 | -0.25 | 0.048 | -0.19 | 0.127 |
| KIRC | -0.047 | 0.281 | 0.11 | 0.012 | 0.024 | 0.585 |
| KIRP | -0.062 | 0.297 | 0.097 | 0.101 | 0.004 | 0.939 |
| LAML | -0.01 | 0.904 | 0.086 | 0.294 | 0.029 | 0.721 |
| LGG | -0.18 | <0.001 | -0.12 | 0.004 | -0.16 | <0.001 |
| LIHC | -0.004 | 0.933 | -0.15 | 0.003 | -0.067 | 0.198 |
| LUAD | -0.05 | 0.244 | -0.11 | 0.015 | -0.089 | 0.041 |
| LUSC | -0.19 | <0.001 | -0.1 | 0.023 | -0.15 | 0.001 |
| MESO | 0.003 | 0.98 | -0.03 | 0.784 | -0.021 | 0.846 |
| OV | -0.14 | 0.007 | -0.023 | 0.652 | -0.095 | 0.064 |
| PAAD | -0.11 | 0.16 | -0.16 | 0.036 | -0.14 | 0.055 |
| PCPG | 0.25 | 0.001 | 0.27 | <0.001 | 0.27 | <0.001 |
| PRAD | 0.068 | 0.127 | 0.006 | 0.885 | 0.034 | 0.447 |
| READ | -0.035 | 0.651 | -0.059 | 0.445 | -0.044 | 0.569 |
| SARC | -0.015 | 0.804 | 0.19 | 0.002 | 0.062 | 0.313 |
| SKCM | -0.032 | 0.493 | -0.033 | 0.479 | -0.036 | 0.439 |
| STAD | -0.23 | <0.001 | -0.3 | <0.001 | -0.3 | <0.001 |
| TGCT | -0.36 | <0.001 | -0.03 | 0.706 | -0.3 | <0.001 |
| THCA | 0.48 | <0.001 | 0.53 | <0.001 | 0.53 | <0.001 |
| THYM | -0.063 | 0.497 | 0.15 | 0.115 | -0.009 | 0.925 |
| UCEC | 0.065 | 0.125 | 0.064 | 0.131 | 0.068 | 0.108 |
| UCS | -0.082 | 0.548 | 0.11 | 0.438 | 0.024 | 0.861 |
| UVM | 0.21 | 0.068 | 0.23 | 0.038 | 0.22 | 0.049 |

| **Table S5 Correlation between PCSK9 expression and PDCD1 expression, CD274 expression and CTLA4 expression in different cancers.** | | | | | | |
| --- | --- | --- | --- | --- | --- | --- |
| **Cancer types** | **PDCD1** | | **CD274** | | **CTLA4** | |
|  | **cor** | ***P*-value** | **cor** | ***P*-value** | **cor** | ***P*-value** |
| ACC | -0.031 | 0.783 | 0.144 | 0.205 | -0.077 | 0.499 |
| BLCA | 0.23 | *** | 0.352 | *** | 0.252 | *** |
| BRCA | 0.146 | *** | 0.173 | *** | 0.154 | *** |
| BRCA-Basal | 0.032 | 0.711 | 0.047 | 0.582 | 0.104 | 0.219 |
| BRCA-Luminal | 0.114 | ** | 0.171 | *** | 0.06 | 0.139 |
| BRCA-Her2 | 0.134 | 0.279 | 0.123 | 0.319 | 0.197 | 0.11 |
| CESC | -0.147 | * | 0.038 | 0.51 | -0.083 | 0.148 |
| CHOL | -0.109 | 0.527 | -0.167 | 0.329 | -0.271 | 0.11 |
| COAD | 0.178 | *** | 0.106 | * | 0.106 | * |
| DLBC | 0.352 | * | 0.266 | 0.068 | 0.23 | 0.116 |
| ESCA | -0.095 | 0.197 | 0.051 | 0.491 | -0.019 | 0.794 |
| GBM | 0.068 | 0.403 | 0.088 | 0.28 | 0.056 | 0.493 |
| HNSC | -0.086 | * | -0.111 | * | -0.127 | ** |
| KICH | -0.021 | 0.866 | -0.292 | * | -0.046 | 0.712 |
| KIRC | -0.079 | 0.068 | -0.05 | 0.251 | -0.018 | 0.68 |
| KIRP | 0.191 | ** | 0.113 | 0.055 | 0.131 | * |
| LGG | -0.103 | * | 0.112 | * | 0.002 | 0.971 |
| LIHC | 0.154 | ** | 0.078 | 0.131 | 0.169 | ** |
| LUAD | -0.062 | 0.157 | -0.135 | ** | -0.096 | * |
| LUSC | -0.133 | ** | -0.076 | 0.089 | -0.106 | * |
| MESO | -0.128 | 0.238 | -0.041 | 0.705 | -0.061 | 0.571 |
| OV | -0.077 | 0.181 | 0.003 | 0.953 | -0.112 | 0.052 |
| PAAD | -0.053 | 0.485 | -0.06 | 0.421 | -0.024 | 0.745 |
| PCPG | 0.129 | 0.084 | -0.015 | 0.846 | 0.209 | ** |
| PRAD | 0.223 | *** | 0.154 | *** | 0.081 | 0.069 |
| READ | 0.1 | 0.2 | 0.057 | 0.467 | 0.031 | 0.693 |
| SARC | -0.049 | 0.431 | -0.185 | ** | -0.016 | 0.798 |
| SKCM | -0.049 | 0.286 | -0.039 | 0.396 | 0.061 | 0.184 |
| STAD | -0.115 | * | 0.094 | 0.056 | -0.01 | 0.832 |
| TGCT | -0.391 | *** | -0.251 | ** | -0.394 | *** |
| THCA | 0.188 | *** | 0.218 | *** | 0.471 | *** |
| THYM | -0.104 | 0.256 | 0.266 | ** | 0.059 | 0.523 |
| UCEC | 0.062 | 0.15 | 0.109 | * | 0.112 | ** |
| UCS | -0.018 | 0.894 | -0.171 | 0.202 | 0.17 | 0.207 |
| UVM | 0.212 | 0.059 | 0.322 | ** | 0.301 | ** |
| ns *P ≧* 0.05; **P* < 0.05; ***P* < 0.01 ; ****P* < 0.001. | | | | | | |
